# Quantitative PCR-Based Analysis of Bacterial Profiles in Periapical Lesions and Maxillary Sinus in Odontogenic Sinusitis

**DOI:** 10.3390/ijms27094010

**Published:** 2026-04-30

**Authors:** Marta Aleksandra Kwiatkowska, Alicja Trębińska-Stryjewska, Katarzyna Andrejuk, Dariusz Jurkiewicz, Elżbieta Anna Trafny, Aneta Guzek

**Affiliations:** 1 Department of Otolaryngology and Oncological Laryngology with Division of Cranio-Maxillo-Facial Surgery, Military Institute of Medicine–National Research Institute, 04-141 Warsaw, Poland; 2 Biomedical Engineering Centre, Institute of Optoelectronics, Military University of Technology, 00-908 Warsaw, Poland; alicja.trebinska@wat.edu.pl (A.T.-S.); katarzyna.andrejuk@wat.edu.pl (K.A.); elzbieta.trafny@wat.edu.pl (E.A.T.); 3 Department of Laboratory Diagnostics, Section of Microbiology, Military Institute of Medicine–National Research Institute, 04-141 Warsaw, Poland; aguzek@wim.mil.pl

**Keywords:** odontogenic sinusitis, polymerase chain reaction, endoscopic sinus surgery, periapical lesion, next generation sequencing, oroantral communication

## Abstract

Odontogenic sinusitis (ODS) is a common cause of unilateral maxillary sinusitis arising from periapical lesions (PALs) or other dental sources. The infection is typically polymicrobial and dominated by anaerobic bacteria, which are often under detected by routine culture. Molecular approaches such as quantitative polymerase chain reaction (QPCR) and next-generation sequencing (NGS) may provide improved characterization of the microbial burden and community structure. This study aimed to compare culture-based methods, targeted quantitative PCR, and 16S rRNA sequencing in paired samples to characterize microbial composition of ODS and evaluate diagnostic performance. Paired sinus mucosal biopsy (SIN) and periapical lesion (PAL) samples were collected from 28 patients with clinically confirmed ODS. Bacterial detection was performed using conventional culture and targeted QPCR assays for ten clinically relevant taxa. In three randomly selected patients, paired samples were additionally analyzed by 16S rRNA gene amplicon sequencing. Microbial load, taxa richness, and similarity between the two anatomically connected sites were assessed using Wilcoxon signed-rank, McNemar, Jaccard distance, and Bray–Curtis dissimilarity analyses. Results: Culture showed low sensitivity, identifying a limited number of pathogens, primarily *Staphylococcus aureus*, *Streptococcus anginosus*, and *Fusobacterium nucleatum*, in a minority of samples. In contrast, QPCR demonstrated substantially higher detection rates, particularly in PAL samples. *Porphyromonas gingivalis* (96.8%), *Fusobacterium* spp. (100.0%), and the *S. anginosus* group (90.3%) were highly prevalent in PAL specimens, with overlapping but lower detection in SIN samples. PAL samples exhibited significantly higher bacterial loads and taxa richness than paired SIN samples (Wilcoxon *p* = 0.0004). 16S rRNA sequencing confirmed polymicrobial communities at both sites and identified additional taxa not included in the QPCR panel. Similarity analyses revealed pronounced interindividual variability, ranging from near-identical to highly divergent paired microbiota. Periapical lesions act as reservoirs of predominantly anaerobic bacteria that may seed the maxillary sinus in ODS. Although microbial overlap exists, sinus communities display lower burden and site-specific compositional shifts. Culture-based diagnostics underestimate ODS microbial complexity, whereas combined molecular approaches provide a more comprehensive and clinically informative assessment.

## 1. Introduction

Odontogenic sinusitis (ODS) is a distinct form of primarily maxillary sinusitis, arising from dental infections like periodontitis, periapical lesions, and complications of dental procedures such as extractions or implant placements [1,2,3]. Unlike rhinogenic chronic sinusitis (CRS), which is primarily associated with viral or allergic etiologies, ODS is often polymicrobial, with a predominance of anaerobic bacteria originating from the oral cavity [4,5,6]. Understanding the microbial load and composition in ODS is crucial for accurate diagnosis and effective treatment, particularly in an era of rising antimicrobial resistance [7,8,9]. ODS can also serve as a portal for systemic dissemination of oral pathogens, which have been implicated in complications such as orbital cellulitis, intracranial abscess, and bacteremia, with life-threatening consequences [10,11,12].

Traditional culture-based microbiological methods have long been the gold standard for identifying bacterial pathogens in clinical samples. However, culture techniques are inherently limited in detecting fastidious and anaerobic bacteria, which are frequently implicated in odontogenic infections [4,9]. As a result, culture-based studies likely underestimate both the bacterial burden and compositional complexity of odontogenic infections, limiting their utility for comparative microbial analyses across anatomically connected sites. Without proper microbial identification, treatment failures and chronicity are common, necessitating repeated interventions [4,8]. To date, international European guidelines recommend antibiotic treatment for sinusitis without considering the potential underlying odontogenic cause [2]. Specific antibiotics that target mainly the upper respiratory tract pathogens responsible for ‘non-odontogenic’ sinusitis might not be adequate for the anaerobe-dominated microbial profile of ODS. This limitation has driven interest in molecular techniques, such as polymerase chain reaction (PCR) and next-generation sequencing (NGS), which offer enhanced sensitivity and broader microbial profiling capabilities [8,13,14,15].

PCR allows for the targeted detection of bacterial species based on their specific genetic markers, such as fragments of the 16S rRNA gene, often enabling the identification of pathogens that are difficult to culture [16]. In contrast, 16S rRNA amplicon sequencing provides a comprehensive overview of the entire microbial community within a sample, allowing for the characterization of both culturable and unculturable bacteria, as well as the assessment of microbial diversity and abundance [8]. Despite these advantages, molecular methods based on microbial DNA targeting present their own challenges, including potential detection of non-viable organisms and susceptibility to environmental contamination [14,17].

Recent studies have highlighted the limitations of conventional culture techniques in detecting the full spectrum of bacteria present in odontogenic infections. For instance, a study of purulent aspirate from the sinus cavity and sequencing the V1-V2 regions of the 16S rRNA gene identified a predominantly polymicrobial spectrum with many genera of anaerobic bacteria, notably *Fusobacterium, Prevotella*, and *Porphyromonas*, associated with periodontal disease [18,19]. This underscores the importance of precise microbiological diagnostics in managing odontogenic abscesses. Another investigation revealed that the sequencing of the 16S rRNA gene hypervariable regions V1-V3 detected considerably more bacterial taxa than conventional cultural methods, even in culture-negative samples, emphasizing the need for updated diagnostic practices [20,21].

A comparative analysis of culture, PCR, and 16S rRNA amplicon sequencing results in clinically confirmed ODS has not yet been done. Similarly, studies are needed that directly compare the microbial communities of ODS in the samples taken from the maxillary sinus (MS) and corresponding periapical lesions. This comparison would help elucidate the microbial continuum between periapical infections and sinus involvement. One approach for this analysis could involve examining restricted-length DNA fragments of the chosen pathogens using pulsed-field electrophoresis. However, it is important to note that such a comparison would focus only on culturable and well-grown bacterial strains, which are typically aerobic and belong to just one species. To compare the numerous species present in these two anatomical sites, whose natural separation is disrupted by infection, we employed culture-based microbiological methods and compared them to two molecular techniques: targeted PCR and NGS. We sought to evaluate the efficiency of detecting pathogenic bacterial taxa using these methods. Candidate pathogens for PCR were selected through an extensive literature review, which identified taxa most strongly implicated in ODS [22,23,24] and the ones more likely associated with non-odontogenic CRS, such as *Pseudomonas aeruginosa* and *Staphylococcus aureus* [22]. We designed primers accordingly and performed both qualitative and quantitative analyses with targeted PCR. For three patients, we additionally analyzed material from both sites using 16S rRNA amplicon sequencing.

This study aimed to compare culture-based methods, targeted quantitative PCR, and 16S rRNA sequencing in paired periapical lesion and MS samples in patients with odontogenic sinusitis, to characterize microbial composition and evaluate diagnostic performance.

Our results showed that conventional culture was less effective than molecular techniques at capturing the full range of bacteria present in ODS infections and were also biased towards aerobic taxa. QPCR and 16S rRNA amplicon sequencing revealed a high degree of heterogeneity within the ODS patient group, with strong overlap between the bacterial communities of the MS and the corresponding periapical lesions in some patients, and discordant results in others. A comparison of QPCR and 16S rRNA amplicon sequencing revealed varying degrees of correlation. More concordant results were obtained for sinus mucosal biopsies than for samples from periapical lesions, demonstrating the suitability of the taxa selected for the QPCR analysis, which targeted ODS-associated bacteria present in the MS. However, in one out of the three patients analyzed by NGS, ten pathogenic taxa chosen for targeted PCR failed to capture the diversity, representing only a small fraction of the taxa detected in sinus biopsies. These findings suggest that although targeted PCR may be a rapid, cost-effective and clinically valuable option, deeper analysis with NGS to obtain a complete microbiological profile should be considered in patients who do not respond to treatment guided by targeted PCR results. Although our study involved a relatively small cohort, it was prospective in nature and highlights the need for further research into the diagnosis of ODS.

## 2. Results

A total of 28 participants were included in the analysis. The mean age of the study population was 48.32 ± 14.40 years. The mean body mass index was 26.56 ± 5.67 kg/m^2^, indicating that, on average, participants were within the overweight range.

Patient-reported outcome measures demonstrated a mean Oral Health Impact Profile score of 10.82 ± 11.23, reflecting substantial variability in perceived oral health–related quality of life. The mean Sinonasal Outcome Test score was 35.50 ± 24.15, indicating a wide distribution of sinonasal symptom severity across the cohort.

Endoscopic evaluation revealed a mean nasal polyps score of 0.18 ± 0.39. The mean discharge score was 1.43 ± 0.63, while mucosal swelling demonstrated a mean value of 0.93 ± 0.26. MS patency showed a mean of 1.86 ± 0.36, suggesting that partial or complete obstruction was frequently observed.

Radiological assessment demonstrated a mean Zinreich score of 4.21 ± 1.26 and a mean Lund–Mackay score of 6.21 ± 2.38, consistent with moderate overall sinus involvement. Regional sinus scores revealed mean values of 1.75 ± 0.44 for the MS, 1.32 ± 0.55 for the frontal ethmoids, 0.57 ± 0.74 for the posterior ethmoids, 0.68 ± 0.77 for the frontal sinus, and 0.25 ± 0.44 for the sphenoid sinus, indicating heterogeneous distribution of disease across sinus compartments.

Dental assessment demonstrated a mean Estrela scale score of 3.75 ± 0.70. Bone expansion or destruction was observed with a mean value of 1.75 ± 0.44. Previous endodontic treatment was present with a mean value of 0.64 ± 0.49, indicating that a substantial proportion of participants had a history of endodontic intervention.

The mean Oral Hygiene Index–Simplified score was 2.96 ± 1.46. The data of the clinical variables of all included patients are presented in Supplementary Table S1.

Conventional culture detected nine pathogenic bacterial taxa in 31 tooth socket swabs and 28 ipsilateral MS irrigation samples from 28 ODS patients (Table 1 and Table S4). Pathogens such as *S. aureus*, *S. anginosus*, and *Fusobacterium* spp. were recovered; however, the overall detection rate was low, with many samples yielding no culturable pathogens. Furthermore, there was only a small overlap in the taxa between tooth socket swabs and MS irrigation isolates, reflecting the restricted range of taxa detectable by culture in ODS samples.

On the other hand, quantitative PCR (QPCR) detected a substantially broader range of bacterial taxa than culture (Table 2 and Table S5). High prevalence rates were observed in tissue scrapings from the periapical lesion (PAL) for *P. gingivalis* (96.8%), *Fusobacterium* spp. (100.0%), and the *S. anginosus* group (90.3%). Although sinus mucosal biopsy specimens (SIN) were less frequently positive, they still showed notable detection rates for the same taxa, with rates 89.3%, 67.9%, and 50.0%, respectively. Additionally, *E. corrodens*, *P. endodentalis*, and *P. nigrescens* were frequently detected in both PAL and SIN samples in several instances, with all three taxa more frequent in PAL than in SIN samples. In contrast, some species such as *P. anaerobius* and *S. aureus* were rarely detected or completely absent.

The calculated 95% confidence intervals (CI) for the 10 bacterial taxa largely aligned with the study’s initial precision assumption of a 20% margin of error. For species with a prevalence near 50%, such as *P. endodontalis* in the sinus (48.1%, 95% CI: 30.7–66.0%), the interval width (35.3%) remained within the expected bounds of the exploratory design. Conversely, for highly prevalent taxa like *p. gingivalis* and *Fusobacterium*, the intervals were notably narrower, providing higher-than-anticipated precision.

QPCR was applied to the full study cohort, while 16S rRNA amplicon sequencing was performed in a small subset of paired samples with sufficient DNA (*n* = 3), together providing complementary information on the microbial composition of SIN and PAL samples.

**Figure 1:**
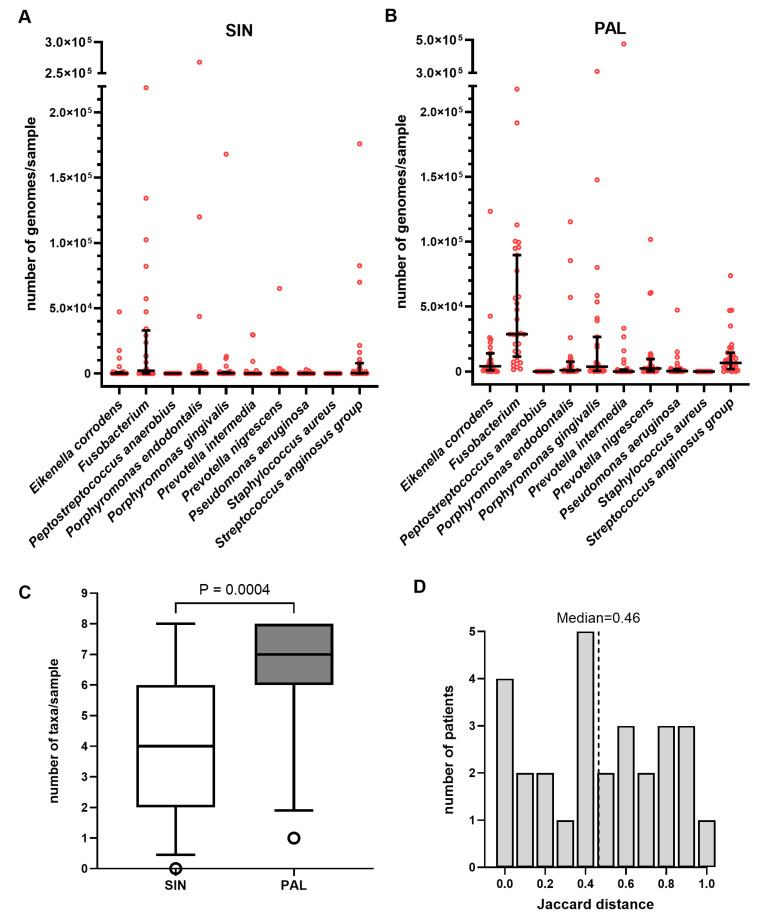
Quantitative comparison of bacterial load, diversity, and site similarity in ODS patients. Bacterial profiles were determined via QPCR using 16S rRNA-specific primers for 10 targeted taxa. Results were quantified using standard curves generated from reference bacterial DNA. (**A**,**B**) Total bacterial genome counts per sample in (**A**) sinus biopsies (SIN) and (**B**) periapical lesions (PALs). Data points represent individual values; horizontal lines indicate the median with interquartile range (IQR). (**C**) Taxonomic richness per sample. The box plot displays the median with IQR; whiskers represent the 5th and 95th percentiles, with open circles denoting outliers. Taxonomic counts between paired SIN and PAL samples were compared using the Wilcoxon matched-pairs test (*p* = 0.0004). (**D**) Microbial similarity between SIN and PAL samples per patient expressed as Jaccard distance. The histogram shows the distribution of distances, where 0 represents identical bacterial composition and 1 represents total dissimilarity. A Wilcoxon signed-rank test confirmed that Jaccard distances were significantly greater than zero (*p* < 0.0001). Note: *Streptococcus anginosus* group includes *S. anginosus*, *S. constellatus*, and *S. intermedius*.

Quantitative PCR demonstrated substantial bacterial loads in both study sites, with bacterial genome counts consistently higher in PAL specimens compared to paired SIN samples (Figure 1A,B, Supplementary Table S5). This increased burden in the dental site was accompanied by greater taxonomic diversity; the number of detected taxons was significantly higher in PAL than in the corresponding SIN samples (Wilcoxon matched-pairs test, *p* = 0.0004; Figure 1C). In the periapical lesions, *Fusobacterium* spp. reached the highest median bacterial load, followed by the *S. anginosus* group, *E. corrodens*, and *P. gingivalis* (Figure 1B). While *Fusobacterium* spp. remained the most dominant taxon within the sinus, it was detected at lower absolute loads along with the *S. anginosus* group, *P. gingivalis*, and *P. endodontalis* (Figure 1A).

The similarity between paired SIN and PAL microbial profiles showed notable variation across the cohort. Jaccard distance analysis, based on the presence or absence of the ten target bacteria, revealed a median distance of 0.46 (Figure 1D). This statistical measure was used to quantify the dissimilarity between paired samples, where a value of 0.00 represents identical bacterial composition and a value of 1.00 indicates that the SIN and PAL sites share no overlapping taxa. While several patients exhibited identical microbial profiles between sites (e.g., P9 and P27; distance 0.00), others demonstrated total dissimilarity (e.g., P2; distance 1.00). Despite these individual instances of concordance, Wilcoxon signed-rank testing confirmed that the microbial community structures between the sinus and periapical sites were significantly different overall (*p* < 0.0001).

**Figure 2:**
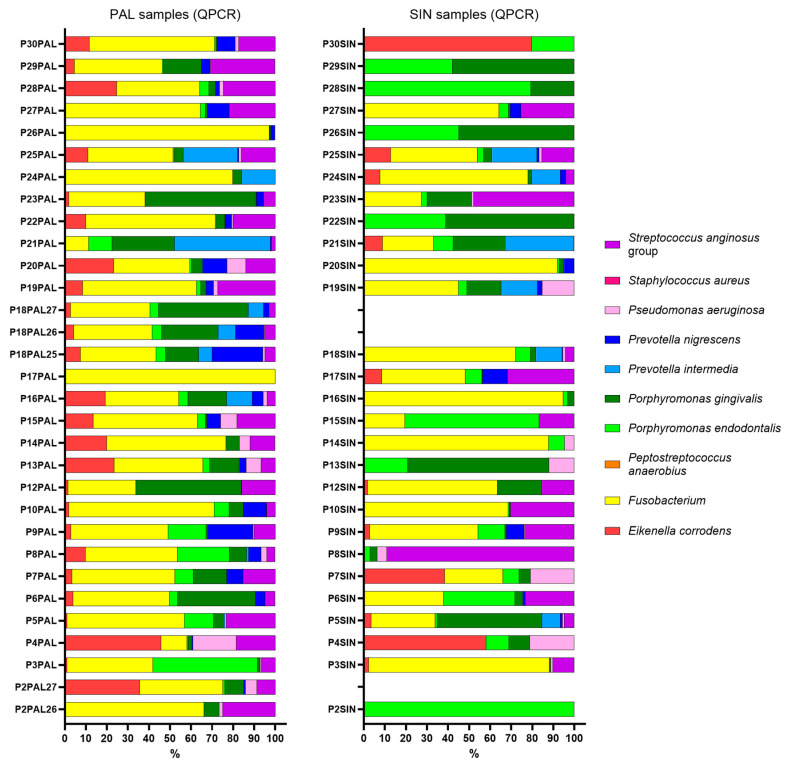
Taxonomic distribution and relative abundance of the targeted bacterial community. Comparison of the relative abundance of 10 selected bacterial taxa detected by QPCR in matched sinus biopsy specimens (SIN) and periapical lesion samples (PAL) from ODS patients. Data illustrate the proportional contribution of each species to the total targeted bacterial load at both sites. Individual colors represent the ten specific bacterial taxa as defined in the legend. Note: *Streptococcus anginosus* group includes *S. anginosus*, *S. constellatus*, and *S. intermedius*.

To further compare the bacterial profiles obtained by targeted QPCR in SIN and PAL samples, relative abundance estimates were analyzed, as shown in Figure 2 (see also Supplementary Table S5). Relative abundance (%) in QPCR was calculated considering the total number of bacterial genomes from 10 selected taxa listed; the relative abundances of other taxa potentially present in the samples are unknown.

**Figure 3:**
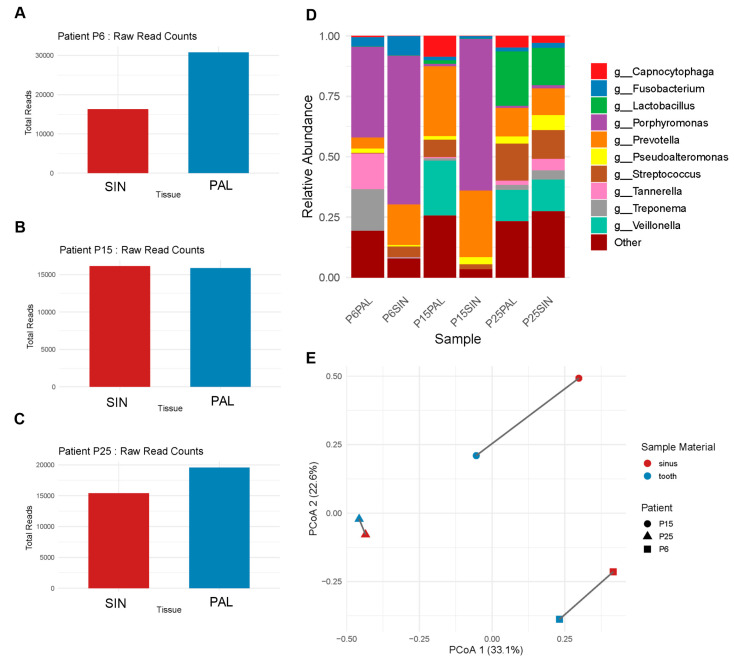
High-resolution taxonomic profiling of matched-pair samples via 16S rRNA amplicon sequencing. Comprehensive microbial analysis was performed on paired SIN and PAL samples from three ODS patients (P6, P15, P25). (**A**–**C**) Total raw sequencing reads obtained for each site in (**A**) Patient 6, (**B**) Patient 15, and (**C**) Patient 25. (**D**) Relative taxonomic abundance at the genus level. Data are normalized to 100% for the top 10 most abundant taxa; the “Other” category encompasses all remaining bacterial genera. (**E**) Principal Coordinates Analysis (PCoA) based on Bray–Curtis dissimilarity, illustrating the microbial community structure. Solid lines connect the paired SIN and PAL samples from each individual patient. The percentages on the axes represent the proportion of total variance explained by the first two principal coordinates (PC1 and PC2).

The 16S rRNA sequencing further illustrated the polymicrobial nature of ODS, revealing complex bacterial communities in both SIN and PAL samples. Sequencing profiles from three patients (P6, P15, P25) highlighted interindividual variability in microbial composition and dominant taxa (Figure 3A–D). Because QPCR targets a predefined set of taxa, relative abundance estimates derived from qPCR are not directly comparable to sequencing-based relative abundances normalized across the entire detected community.

At the genus level, both SIN and PAL samples were dominated by anaerobic genera (e.g., *Prevotella*, *Porphyromonas*), with their relative contributions varying by individual (Figure 3D). When normalized to 100%, the top 10 genera accounted for most reads, while the “Other” category reflected additional microbial diversity (Figure 3D). The most notable discrepancy between the two approaches was the underrepresentation of *Fusobacterium* in the sequencing results, despite its consistent detection by QPCR.

Principal Coordinates Analysis (PCoA) based on Bray–Curtis dissimilarity demonstrated clear clustering of SIN and PAL samples for Patient P25, while also revealing patient-specific variations in microbial composition for Patients P6 and P15 (Figure 3E). Interestingly, these results were concordant with Jaccard distance analysis of QPCR results (P25 = 0.00, P6 = 0.29, P15 = 0.38), despite the broader range of taxa detected in NGS.

These findings indicate that while there is significant overlap between the microbiota of the periapical area and the sinuses in cases of odontogenic sinusitis, the microbial community in these two anatomical sites are not identical. It also shows a distinct enrichment of certain anaerobic species. This suggests that the microbial composition in the sinus is not simply an extension of the periapical microbiota; rather, it reflects additional ecological pressures and specific selection processes related to the unique environment of the sinus.

Moreover, the use of targeted PCR as a diagnostic method for assessing microbial burden in ODS, compared to 16S rRNA sequencing, suggested potential clinical applicability. In two out of the three patients studied here, the selected primers detected nearly 90% of the bacterial taxa present in the sinus found by 16S rRNA sequencing. It is important to note that these primers were specifically designed based on taxa commonly found in sinus samples, rather than those from the dental microbiota of patients with ODS. This distinction is clearly reflected in our findings. However, in the third patient, whose sinus and dental microbiota were highly similar, the primers designed for sinus-dominant taxa detected less than 30% of the bacterial burden in the sinus samples. These results emphasize the significant diversity of microbiomes in ODS patients and highlight the diagnostic challenges associated with selecting the most appropriate technique.

## 3. Discussion

Despite being a well-established clinical entity, ODS is less characterized compared to other subtypes of chronic rhinosinusitis (CRS), such as CRS with nasal polyps and CRS without nasal polyps [25].

One of the most significant knowledge gaps relates to the microbial etiology of ODS. Most of the existing research on CRS focused on inflammatory mechanisms and microbiota linked to sinonasal dysbiosis, biofilm formation, and host-microbe interactions in the nasal cavity and paranasal sinuses [26,27,28,29]. In contrast, the microbial landscape of ODS is less understood, largely because it originates from a different anatomical and ecological niche: the oral cavity. The translocation of oral pathogens into the maxillary sinuses creates a distinct polymicrobial environment, often dominated by anaerobic species and biofilm-forming microorganisms that are difficult to isolate using standard culture methods [9,30].

Several studies have compared microbial detection techniques, such as culture-based methods, next-generation sequencing (NGS), and polymerase chain reaction (PCR) in CRS diagnostics, but in ODS it remains understudied [4,8,9,23,27,31,32]. Anaerobic bacteria are the predominant microorganisms found in odontogenic lesions, and they exhibit greater diversity than what traditional cultural diagnostics suggest [8]. To date, only a few studies have provided data on microbiome specific for ODS [20,33], and none have analyzed the material using three methods simultaneously: classical microbiology culture, targeted QPCR, and NGS. Due to the logistical challenges and the need for an interdisciplinary approach to ODS patients, existing data have primarily focused on microbial diversity in either sinus or periapical regions [20,33,34,35,36,37,38].

In our QPCR analysis, high bacterial load in PAL samples was detected for *Fusobacterium* spp., *Porphyromonas* spp., *Prevotella* spp., and members of the *S. anginosus* group. These anaerobic and microaerophilic species are typical of periodontal infections and play established pathogenic roles in the spread of infection to deeper tissues [4,10,24,39]. The results are partly consistent with published QPCR analysis on the composition of the microbiota outside the root canal, with the main bacteria identified as *Actinomyces* spp*., Propionibacterium, Prevotella* spp., oral streptococci, *P. endodontalis*, and *Burkholderia* [32,39]. In the literature review by Craig et al. [40] *Prevotella*, *Fusobacterium*, *and Peptostreptococcus* were the most commonly isolated species, although *Peptostreptococcus* was not detected in the cohort described in the presented study. Oral microbiome composition varies across populations and clinical settings, possibly explaining why some cohorts detect *Peptostreptococcus* while others do not. *Fusobacterium nucleatum*, consistently detected at high loads in our QPCR analysis, is considered an organism that facilitates co-aggregation of diverse oral taxa and stabilizes biofilm communities. Its dominance may suppress or ecologically displace *Peptostreptococcus* in some periapical lesions, accounting for the variability in detection across studies [41].

While QPCR demonstrated high specificity for targeted pathogens, 16S rRNA sequencing provided a broader ecological overview, detecting a greater number of taxa. Correlation analysis between the relative abundance of species identified by QPCR and the sequencing technique showed moderate alignment, indicating that both methods may serve complementary roles in microbiological assessment.

A notable discrepancy in our results between QPCR and 16S rRNA amplicon sequencing was the different detection rate of *Fusobacterium* spp. While QPCR consistently identified *Fusobacterium* with high prevalence in both PAL and sinus samples, the sequencing technique frequently underrepresented or classified these reads under broader taxonomic categories. This discrepancy is consistent with previous reports highlighting primer bias and classification limitations in 16S rRNA amplicon sequencing [42,43]. The universal primers used in 16S rRNA amplicon sequencing do not function equally well across all taxa, and *Fusobacterium* often exhibit mismatches at conserved primer-binding regions, leading to reduced amplification. Furthermore, taxonomic assignment pipelines can misclassify *Fusobacterium* reads into higher-level categories, particularly when sequence similarity is high within oral anaerobe clades. In contrast, the group-specific QPCR method used in this study ensured targeted and sensitive detection and quantification even at low abundance levels. Nevertheless, the underrepresentation of *Fusobacterium* in the NGS data cannot be attributed solely to primer bias. Previous studies have reported reduced sensitivity of V3–V4 regions for certain anaerobic taxa, including *Fusobacterium* spp. Alternative approaches, such as targeting V1–V3 regions or employing shotgun metagenomic sequencing, may provide improved taxonomic resolution [41].

The current hypothesis suggests that ODS arises from the translocation of pathogenic bacteria from periapical lesions into the MS. This process leads to a distinct yet overlapping microbial community between the two sites. While PAL samples contain a higher bacterial burden and greater microbial diversity, certain anaerobic species are consistently found in both the sinus microbiota and the PAL samples. The sinus environment further modifies this microbial composition, leading to selective enrichment or depletion of particular taxa [44,45]. Our study corroborated the finding of greater microbial diversity in PAL compared to SIN samples. QPCR analysis revealed a significantly higher number of selected taxa in PAL samples (Wilcoxon test: *p* = 0.0009). Furthermore, 16S rRNA amplicon sequencing revealed greater microbial diversity in the analyzed PAL samples. Comparison of microbiota from the dental socket (post-extraction site) and MS using Jaccard distance calculation revealed significant differences between these sites (*p* < 0.0001). While some patients exhibited nearly identical microbial profiles, others showed only partial overlap and individualized patterns of bacterial colonization. These results emphasize the importance of dual-site sampling to capture the full microbiological landscape of ODS. The bacterial communities detected within the MS mucosa appear to reflect a selective ecological environment rather than a passive extension of the oral microbiota. Despite anatomical proximity to odontogenic sources, sinus-associated bacterial profiles showed limited representation of aerobic taxa, including organisms such as *Staphylococcus aureus*, which are frequently recovered by culture but were inconsistently detected by molecular methods in sinus samples [17]. This observation is consistent with prior evidence indicating that the sinus milieu favors low-oxygen–adapted organisms and may actively constrain the persistence of obligate aerobes or facultative taxa that are not well suited to this environment [32,46,47]. Notably, taxa known for biofilm-forming capacity and anaerobic metabolism were more consistently identified, supporting the concept that adherence, interbacterial cooperation, and tolerance to hypoxic conditions may contribute to bacterial persistence within the sinus mucosa [30]. Together, these findings support a model in which the MS functions as a relatively stable ecological niche that selectively permits colonization by specific bacterial groups, rather than a compartment characterized by unrestricted microbial translocation or random variability. Findings point toward a more dynamic microbial exchange between dental and sinonasal environments, which is influenced by factors such as host immunity, mucosal barrier function, and environmental conditions like oxygen availability [44].

Importantly, molecular profiling has implications for treatment. The high prevalence of anaerobes and mixed infections identified through molecular methods suggests that empiric antibiotic regimens used in CRS may be suboptimal for ODS. This condition often requires antibiotics that specifically target oral anaerobes, such as *Fusobacterium*, *Prevotella*, and *Porphyromonas*. Therefore, molecular diagnostics may serve as a basis for personalized therapeutic strategies, especially in cases that are recurrent or refractory to standard therapies.

Although 16S rRNA sequencing provided the most comprehensive insight into the microbial diversity of ODS, it is expensive and requires advanced technical and bioinformatic expertise, which may limit its routine application in smaller hospital laboratories. Classical microbial culture proved to be the least effective method here, mainly due to the anaerobic physiology of the predominant pathogens and the diagnostic challenges associated with this. However, without pure isolates, the detection of multidrug-resistant strains remains impossible unless whole-genome sequencing is performed, which further increases both the cost and workload.

Our results showed that conventional culture was less effective than molecular techniques at capturing the full range of bacteria present in ODS infections and were also biased towards aerobic taxa. QPCR and 16S rRNA amplicon sequencing revealed a high degree of heterogeneity within the ODS patient group, with strong overlap between the bacterial communities of the MS and the corresponding PAL in some patients, and discordant results in others. A comparison of QPCR and 16S rRNA amplicon sequencing revealed varying degrees of correlation. More concordant results were obtained for sinus mucosal biopsies than for samples from periapical lesions, demonstrating the suitability of the taxa selected for the QPCR analysis, which targeted ODS-associated bacteria present in the MS. However, in one out of the three patients analyzed by NGS, ten pathogenic taxa chosen for targeted PCR failed to capture the diversity, representing only a small fraction of the taxa detected in sinus biopsies. These findings suggest that although targeted PCR may be a rapid, cost-effective and clinically valuable option, deeper analysis with NGS to obtain a complete microbiological profile should be considered in patients who do not respond to treatment guided by targeted PCR results. Although our study involved a relatively small cohort, it was prospective in nature and highlights the need for further research into the diagnosis of ODS.

## 4. Materials and Methods

### 4.1. Study Setting, Design and Participants

The sample size was determined based on the requirements for an exploratory study, with the primary objective of estimating the prevalence of 10 specific bacterial taxa in paired periapical and sinus samples using the QPCR method. To ensure the most conservative estimate for exploratory research, the sample size was calculated using the Wald formula for a single proportion: *n = Z*^2^*_α_*_/2_ *× p*(1 − *p*)/*D*^2^. A hypothetical prevalence (*p*) of 0.50 (50%) was assumed, as this value maximizes the required sample size when the true prevalence is unknown. Applying a 95% confidence level (*Z_α_*_/2_ = 1.96) and a desired precision (margin of error) of 20% (*D* = 0.20), the minimum required sample size was determined to be 24 patients. To account for a potential 15% rate of technical failures, such as insufficient DNA yield from samples, the recruitment target was increased to 28 patients. This sample size is consistent with established “rules of thumb” for clinical pilot studies and provides sufficient data to generate descriptive statistics and 95% confidence intervals to inform future, larger-scale investigations.

Adult individuals presenting with both clinical and radiographic features consistent with chronic odontogenic sinusitis (ODS), accompanied by periapical lesions (PALs) associated with maxillary premolars or molars, were eligible for inclusion in this study. Participants were recruited from the inpatient service of the Department of Otolaryngology with the Division of Cranio-Maxillo-Facial Surgery at a tertiary referral center.

Each participant underwent a comprehensive assessment conducted jointly by an otolaryngologist and a dental specialist. The otolaryngological evaluation included a detailed medical history, nasal endoscopic examination, and radiological imaging using computed tomography (CT) or cone-beam computed tomography (CBCT) of the paranasal sinuses and adjacent maxillary dentition. CT was considered the primary imaging modality for the assessment of sinonasal disease and surgical planning. However, in patients referred with high-quality CBCT scans that provided adequate visualization of both the sinuses and adjacent dentition, additional CT imaging was not performed in order to avoid unnecessary radiation exposure. Radiographic evidence of sinusitis was defined as mucosal alterations within the ostiomeatal complex and/or paranasal sinuses exceeding 50% of the sinus cavity.

Dental examination of periapical tissues comprising percussion and palpation testing, assessment of tooth mobility, pulp vitality testing using cold stimuli, and detailed radiographic analysis based on CT or CBCT imaging.

The following demographic and clinical parameters were collected: age, sex, body mass index (BMI), laterality of ODS, sinonasal symptom profile, and nasal endoscopic findings including purulence, mucosal edema, and the presence of polyps, evaluated according to a modified Lund–Kennedy scoring system at baseline or at the time of surgery. Radiological assessment included the extent of sinus opacification on CT imaging. Dental variables encompassed the presence and characteristics of periapical pathology with the use of the Estrela scale, history of endodontic treatment, and patient-reported dental symptoms. Oral hygiene status was assessed using the Oral Hygiene Index–Simplified (OHI-S), calculated as the sum of the Debris Index and Calculus Index scores obtained from six representative tooth surfaces. Sampling from both sinus and periapical sites was performed intentionally, including in cases of anatomical continuity, to evaluate potential microbiological differences between compartments.

Exclusion criteria included patients with bilateral sinus involvement accompanied by bilateral periapical lesions, suspected coexistence of fungal ball disease with ODS, and individuals with primary immunodeficiency disorders. Patients presenting solely with reversible dental conditions or isolated mucosal thickening of the MS without clear odontogenic origin were also excluded from the analysis. The detailed clinical data are given in Supplementary Table S1.

### 4.2. Sample Collection

Samples for the microbiological, quantitative QPCR, and 16S rRNA sequencing analysis were collected at the beginning of ESS, coinciding with the extraction of the causative tooth. To minimize cross-contamination, sinus and dental samples were collected using separate sterile instruments, and sinus sampling was performed prior to dental manipulation. After removing the horizontal part of the uncinate process, small maxillary antrostomy was done. Then, a curved suction was introduced into the ostium of MS and the purulent fluid was suctioned out from its lumen and immediately stored in a sterile container. A mucosal biopsy was then taken from the floor of the MS using curved forceps. Then the surgery was carried out with widening the MS opening. In the next step, following the extraction of the causative tooth, a sterile swab was collected from the tooth socket with a periapical lesion, and tissue scrapings were obtained from curettage of the remaining periapical lesion. The MS discharge and tooth socket swabs were immediately sent for culturing to the Department of Laboratory Diagnostics.

Separate sterile instruments were used for sinus and dental sampling, with sinus samples collected prior to dental manipulation.

Samples from sinus mucosal biopsy (SIN) and the tissue scrapings from periapical lesion (PAL) were immediately stored in separate 1 mL vials with DNAgard Tissue and Cells (^®^Biomatrica, San Diego, CA, USA) sterile solution and stored at 4 °C.

### 4.3. Microbiological Analysis

The culture and bacterial species identification were performed in accordance with routine diagnostic procedures at the Department of Laboratory Diagnostics, Military Institute of Medicine–National Research Institute. The material was promptly cultured on nonselective media (Columbia agar supplemented with 5% sheep blood, McConkey agar, Chapman agar, and Sabouraud agar), as well as the selective medium for *Haemophilus* species, and Schaedler agar for the cultivation of anaerobic bacteria. All plates were incubated for 24–48 h at 37 °C under a 5% CO_2_ atmosphere, except for Schaedler agar plates, which were cultured at the same temperature but under anaerobic conditions. The isolated colonies were accurately identified using Matrix-Assisted Laser Desorption Ionization-Time of Flight mass spectrometry (MALDI-TOF) (VITEK MS, bioMérieux, France) according to the manufacturer’s instructions.

### 4.4. DNA Isolation

Prior to DNA isolation, PAL and SIN samples were sonicated twice for 30 s at room temperature in an ultrasonic washer (Polsonic, Warsaw, Poland). Freshly prepared lysozyme (100 µL, stock 100 mg/mL, Thermo Fisher Scientific), RNase A (10 µL, from DNA Extraction Genomic Mini, Blirt) and freshly prepared lysostaphin (8 µL, stock 10 mg/mL, Sigma-Aldrich) were added and the samples were incubated for 40 min at 37 °C. DNA was extracted using a DNA Extraction Genomic Mini kit (Blirt, Gdańsk, Poland). Following the addition of GL Lysis Buffer (750 µL) and proteinase K (50 µL), the samples were incubated for 30 min at 55 °C and later transferred to BashingBead Lysis Tubes (Zymo Research). They were vortexed for 5 min at 3000 rpm and subsequently centrifuged for 2 min at 14,500 rpm using an Eppendorf Mini Spin centrifuge. The supernatants were then subjected to on-column DNA purification following the manufacturer’s instructions. DNA was eluted from the columns using 50 µL Elution buffer. Human DNA was not removed from the samples. DNA concentration was determined using QuantiFluor ONE dsDNA System (Promega, Madison, WI, USA) and Quantus Fluorometer (Promega). DNA was aliquoted and frozen at −80 °C until further use.

### 4.5. Primer Design for Quantitative PCR

Primer pairs were designed to amplify the fragments of the 16S rRNA gene. The following bacterial species were selected: *Eikenella corrodens*, *F. nucleatum*, *Peptostreptococcus anaerobius*, *Porphyromonas endodontalis*, *Porphyromonas gingivalis*, *Prevotella intermedia*, *Prevotella nigrescens*, *Pseudomonas aeruginosa*, and *S. aureus*. First, the eHOMD 16S rRNA Reference Sequence Tree from the Human Oral Microbiome Database website [48] (accessed on 20 March 2025) was used to identify the closest relatives of each bacterial species analyzed. Then, the 16S rRNA gene sequences from each group were retrieved from the ATCC Genome Portal [49], NCBI Reference Sequence Database [50,51] and GenBank [51]. Multiple alignments were performed using Clustal Omega with default settings [52,53], and visualized with Jalview version 2.11.1.3 [53]. Regions in which all retrieved 16S rRNA sequences from selected bacterial species were identical and the sequences from their closest relatives exhibited mismatches were selected for primer design with Primer-BLAST [54]. The main Primer-BLAST parameters were as follows: primer length, 15–25 nt; PCR product length, 100–200 bp; primer melting temperature, 57–63 °C (optimal: 60 °C). Primer pairs were screened with Primer-BLAST for self-complementarity and self-3′-complementarity. Each primer pair’s specificity was checked in silico against Bacteria (taxid:2) RefSeq representative genomes, *Homo sapiens* (taxid:9606) reference genome sequence, and the RefSeq human mitochondrial DNA sequence (NC_012920.1). Primers used for the simultaneous detection of *S. anginosus* group (*Streptococcus anginosus, Streptococcus constellatus*, and *Streptococcus intermedius*) 16S rRNA were described previously by Olson et al. [16]. Prior to QPCR on patients’ samples, primers were experimentally tested, and the best-performing primer pairs were used for further experiments. Primer sequences, product lengths, and the results of the in silico specificity analyses are provided in Supplementary Table S2.

### 4.6. Quantitative PCR (QPCR)

Quantitative polymerase chain reaction (QPCR) was employed for absolute quantification. The standard curves were prepared using reference genomic DNA from type strains. Bacterial DNA reference samples were obtained from the German Collection of Microorganisms and Cell Cultures (DSMZ, https://www.dsmz.de/ accessed on 17 August 2021), see Supplementary Table S3. The Brilliant III Ultra-Fast SYBR Green QPCR Master Mix (Agilent, Santa Clara, CA, USA) was used for the QPCR. Each reaction contained 10 µL of 2× SYBR Green QPCR master mix, 30 nM ROX (reference dye), 200 nM of each primer, and 1 µL of DNA template, resulting in a final reaction volume of 20 µL. To generate the standard curves, serial dilutions of the reference DNA were prepared (10 ng/µL, 1 ng/µL, 100 pg/µL, 10 pg/µL, 1 pg/µL, 100 fg/µL, 10 fg/µL), and 1 µL from each dilution was used as the template for the QPCR. To assess the potential for unspecific primer binding to human DNA, genomic DNA from human fibroblasts (1 ng/reaction) was employed. To ascertain the specificity of primer pairs for a particular bacterial species, namely *Prevotella* spp. and *Porphyromonas* spp., genomic DNA (1 ng/reaction) from other species within the same genus was incorporated as a control (results are provided in Supplementary Table S2). Two technical replicates were prepared for each DNA sample. The Agilent Aria Mx instrument was employed, and the QPCR conditions were as follows: a hot start for 3 min at 95 °C, denaturation for 5 s at 95 °C, primer annealing/extension for 10 s at 60 °C, and 45 cycles in total. Following the QPCR, a melt curve analysis was conducted with a resolution of 0.5 °C and a soak time of 5 s.

### 4.7. Quantitative PCR Results Analysis

QPCR results below the lowest point on the standard curve and/or with an incorrect melting curve were excluded from further analysis. DNA mass in each standard curve point was converted into the number of bacterial genomes (Supplementary Table S3). The following data were used for the calculations: genome size in base pairs and GC content retrieved from the ATCC Genome Portal (Supplementary Table S3); GC base pair molecular weight of 616.4 g/mol and a mass of 1.024 × 10^−6^ fg; AT base pair molecular weight of 615.4 g/mol and a mass of 1.022 × 10^−6^ fg. The dissociation of hydrogen ions from phosphate groups in DNA at the physiological pH was accounted for in the calculations [55]. Based on the standard curve, the number of bacterial genomes of each analyzed taxon were calculated for every sample. The results were then converted into percentages of the total.

### 4.8. 16S rRNA Amplicon Library Preparation and Sequencing

Three patients were randomly selected for 16S rRNA amplicon sequencing using a random number generator applied to the full study cohort. These cases were chosen to illustrate methodological differences between raw read counts and relative abundance metrics rather than to support statistical inference. Bacterial 16S rRNA gene fragments were amplified using primers targeting the V3–V4 hypervariable regions, following the standard Illumina 16S Metagenomic Sequencing Library Preparation protocol. Amplicon libraries were prepared using Nextera XT chemistry and sequenced on the Illumina MiSeq platform with a MiSeq Reagent Kit v3 (2 × 300 bp paired-end reads). All steps were performed according to the manufacturer’s instructions. Sequencing was conducted on DNA extracted from PAL and SIN samples collected from three arbitrarily chosen patients diagnosed with odontogenic sinusitis. The same DNA extracts were also used for quantitative PCR analysis.

### 4.9. Bioinformatic Analysis of 16S rRNA Amplicon Sequencing Results

Raw sequencing data were processed using the QIIME 2 pipeline (QIIME2 v. 2023.9) [56]. Demultiplexed reads were denoised and dereplicated using the deblur plugin with default parameters [57]. Taxonomic classification was assigned at the genus level using a pre-trained Naïve Bayes classifier [58] based on the SILVA 16S rRNA gene reference database (SILVA 138.1 SSURef Nr 99) [58]. Prior to beta diversity analysis, the feature table was rarefied to a depth of 10,000 sequences per sample to normalize sequencing depth across samples. Bray–Curtis dissimilarity metrics were calculated, and Principal Coordinates Analysis (PCoA) was performed to visualize differences in microbial community structure. Subsequent statistical analyses and visualizations were conducted in R Studio (version 2024.04.2 + 764) [59] using the phyloseq, dplyr, and ggplot2 packages. The raw sequencing data (FASTQ files) are publicly available in the National Center for Biotechnology Information (NCBI) Sequence Read Archive (SRA) under BioProject accession number PRJNA1333631.

### 4.10. Statistical Analysis of QPCR Results

Data visualization, confidence interval calculations and statistical analysis of QPCR results were performed with GraphPad Prism version 10.3.0 for Windows (GraphPad Software, Boston, MA, USA, www.graphpad.com. Accessed on 5 May 2025. The Wilcoxon matched pairs test was used to compare the number of detected taxa between paired SIN and PAL samples. The Jaccard distance was calculated between paired SIN and PAL samples, based on binary detected/undetected data for each identified bacterial taxon. The Wilcoxon signed-rank test was used to determine whether the Jaccard distances differed significantly from zero. These statistical analyses were performed in Python (3.14.4 Version 2) using *pandas*, *scipy*, and *seaborn* libraries. Jaccard distances were calculated with *scipy.spatial.distance.jaccard*, and the Wilcoxon test was implemented using *scipy.stats.wilcoxon*. A statistical significance level in all tests was set to α = 0.05.

## 5. Limitations

Only three paired SIN and PAL samples underwent 16S rRNA amplicon sequencing. This sample size is insufficient to support population-level conclusions and does not capture the full heterogeneity of ODS-associated microbiomes and should be treated as exploratory. Observed patterns may therefore reflect interindividual variability rather than consistent biological trends.

## 6. Conclusions

QPCR enabled sensitive detection of bacterial taxa in paired periapical and sinus samples, revealing both shared and site-specific microbial patterns not captured by conventional culture. Exploratory 16S rRNA sequencing confirmed the polymicrobial nature of odontogenic sinusitis but was limited in scope. Although overlapping communities were observed, sinus samples demonstrated lower bacterial burden and compositional differences, highlighting interindividual variability. These findings support the added value of molecular approaches, while emphasizing the need for validation in larger, multicenter studies.

## Figures and Tables

**Table 1:** Culturable pathogenic bacteria in ODS patients’ tooth socket swabs (*n* = 31) and ipsilateral sinus irrigation specimens (*n* = 28).

Taxon	% of Positive Tooth Socket Swabs	% of Positive Sinus Irrigation Samples	No. of Paired Positive Tooth Socket Swabs and Sinus Irrigation Samples
*Fusobacterium* spp.	0.0	3.6	0
*Hafnia* spp.	3.2	3.6	1
*Prevotella* spp.	0.0	3.6	0
*Routella* spp.	0.0	3.6	0
*Staphylococcus aureus*	3.2	7.1	1
*Streptococcus anginosus*	0.0	10.7	0
*Streptococcus pneumoniae*	3.2	0.0	0
*Streptococcus pyogenes*	0.0	3.6	0
*Veilonella* spp.	3.2	0.0	0

**Table 2:** Bacterial taxa detected by QPCR in ODS patients’ periapical lesions (PALs, *n* = 31) and sinus biopsy specimens (SIN, *n* = 28).

Taxon	% of Positive PAL Samples [95% CI]	% of Positive SIN Samples [95% CI]	No. of Paired Positive PAL and SIN Samples
*Eikenella corrodens*	83.9 [67.4–92.9]	39.3 [23.6–57.6]	9
*Fusobacterium*	100.0 [89.0–100.0]	67.9 [49.3–82.1]	19
*Peptostreptococcus anaerobius*	0.0 [0.0–11.0]	0.0 [0.0–12.0]	0
Porphyromonas endodontalis *	76.7 [59.1–88.2]	48.1 [30.7–66.0]	12
*Porphyromonas gingivalis*	96.8 [83.8–99.8]	89.3 [72.8–96.3]	24
*Prevotella intermedia*	41.9 [26.4–59.2]	21.4 [10.2–39.5]	5
*Prevotella nigrescens*	77.4 [60.2–88.6]	35.7 [20.7–54.2]	7
Pseudomonas aeruginosa **	88.9 [71.9–96.1]	66.7 [46.7–82.0]	15
*Staphylococcus aureus*	0.0 [0.0–11.0]	3.6 [0.2–17.7]	0
*Streptococcus anginosus* group	90.3 [75.1–96.7]	50.0 [32.6–67.4]	12

* Results for 27 out of 28 patients; ** Results determined for 24 out of 28 patients.

## Data Availability

Newly Generated Data: the Conventional Culture Data was newly generated for this study.

## Supplementary Materials

The following supporting information can be downloaded at: https://www.mdpi.com/article/10.3390/ijms27094010/s1.

## Abbreviations

The following abbreviations are used in this manuscript:

ODS	odontogenic sinusitis
CRS	chronic rhinosinusitis
CT	computed tomography
CBCT	cone-beam computed tomography
PAL	periapical lesion
RCT	root-canal treatment
ESS	endoscopic sinus surgery
MS	maxillary sinus
QPCR	quantitative polymerase chain reaction
OAC	oroantral communication
NGS	next-generation DNA sequencing
SIN	sinus
